# Richard George Worger

**Published:** 1910-03

**Authors:** 


### Obituary Notices.

### RICHARD GEORGE WORGER, M.R.C.S.Eng., L.R.C.P.Lond., L.M.S.S.A.

MR. RICHARD GEORGE WORGER, M.R.C.S.Eng., L.R.C.P.Lond., L.M.S.S.A., died at his residence at Radstock on February 8th, in his thirty-eighth year. He took his L.S.A. in 1895, and his Conjoint diploma in 1896, and had an extensive practice in Radstock and the neighbourhood. He held Poor-Law appointments under the Clutton and Frome Boards of Guardians, was certifying factory surgeon for the district, and surgeon to the Post Office and Great Western Railway employees. He was greatly interested in ambulance work, and was captain in the 2nd S.W. Field Ambulance, R.A.M.C.T. He was an Associate of the Order of St. John of Jerusalem, the Prince of Wales having presented him with the decoration in June, 1909. Mr. Worger was very popular in the district, and much sympathy will be felt for his relatives. The funeral took place on February 11th, with full military honours.

